# Fox on the Run—Cheaper Camera Traps Fail to Detect Fast‐Moving Mesopredators: Assessing the Differences in Mesopredator Detection Efficacy Among Different Camera Trap Models

**DOI:** 10.1002/ece3.70958

**Published:** 2025-02-14

**Authors:** R. McHenry, L. J. Mitchell, C. Marshall, J. Smart, A. L. de Raad, R. Andersen

**Affiliations:** ^1^ Environmental Research Institute, University of the Highlands and Islands Thurso Highland UK; ^2^ RSPB Centre for Conservation Science The Lodge Sandy Bedfordshire UK; ^3^ Game and Wildlife Conservation Trust Aboyne UK; ^4^ Hopetoun Estates Office Hopetoun UK

**Keywords:** camera traps mammals, infrastructure, land use change, predators, roads

## Abstract

Camera trapping for detecting wildlife is increasingly used as a primary method of non‐invasive wildlife monitoring. Yet understanding among researchers and conservationists on how camera trap make, and model affect detection rates is limited. Published studies often fail to make clear why a given camera trap model was chosen or what specifications or parameters were used to capture target species within a given study area, prohibiting replicability. Here we present a comparison of predator and herbivore detection efficacy using three makes and models of camera trap at differing price ranges, year of release (hereafter vintages) and specifications. We used a passive monitoring survey design at six sites in open field conditions across the Flow Country, Northern Scotland. Detection efficacy varied substantially between grades and vintages of camera traps and depended on species captured. Older models of camera with lower trigger speed and night vision range performed particularly poorly for nocturnal predatory mammal detection. This has implications for how researchers, conservationists, developers and other users approach experimental design and analyses, but also on the conclusions that may be drawn from studies. We caution against using the results of one or more camera trap studies using different makes and models of cameras to inform experimental design or policy interventions.

## Graphical abstract

Top left: proportion of deer of total deer images taken in light and darkness for each camera trap type. Top right and bottom images: The same deer event as taken by each camera. Notice differences in FOV and image quality/range.

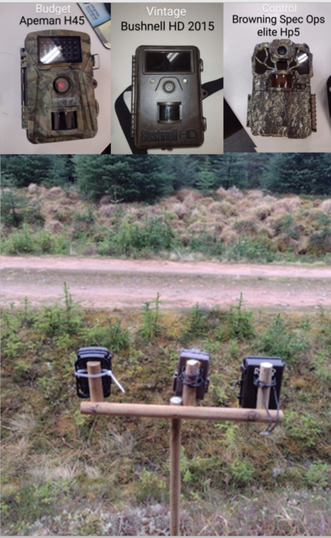

## 1 Introduction

The use of camera traps to monitor biodiversity, particularly the activity and occurrence of large and medium sized, often cryptic, mammals, is continuing to increase in popularity (e.g., Rowcliffe and Carbone 2008; Thapa et al. 2022; Widodo et al. 2022; Salvatori et al. 2023). Relative to other wildlife monitoring methods, camera traps present many advantages in conservation management in that they are non‐invasive, effective across various environmental conditions, are cost and labour efficient and can measure a range of key conservation parameters, including abundance, behaviour, occurrence, community composition and distribution (Meek et al. 2012; Kelly et al. 2012; Swann et al. 2011). Additionally, they allow for continuous monitoring of these factors, which is especially useful for difficult to monitor nocturnal animals. As camera traps have become more affordable and available, methods of survey design and statistical analyses of their data have developed in complexity and efficacy (e.g., Wysong et al. 2022). Researchers can now draw from an extensive literature of camera trap studies to apply robust, tested approaches to experimental design and statistical analyses to answer questions on occupancy, behaviour, and presence in specific experimental and natural contexts (e.g., Ahumada et al. 2020; Kays et al. 2020; Meek et al. 2014; Willi et al. 2019; Wysong et al. 2022).

**FIGURE 1:**
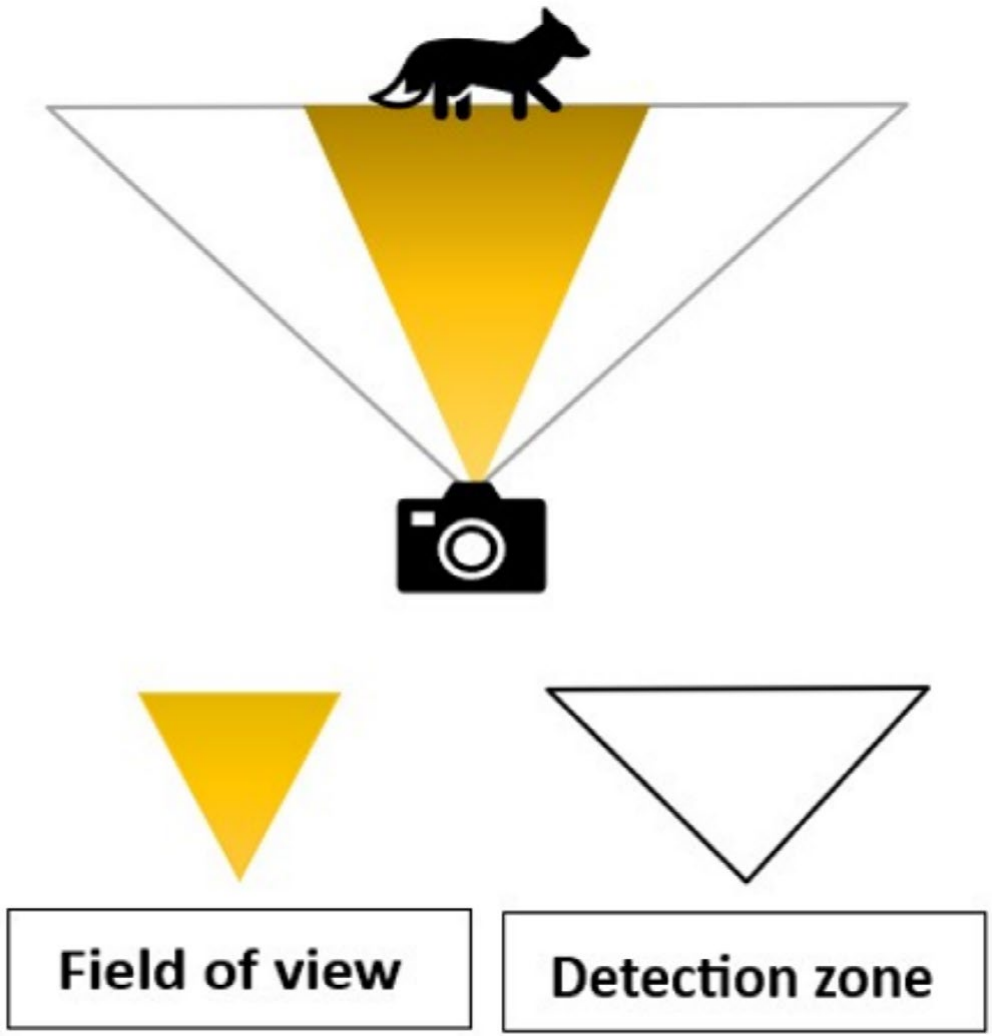
Comparison between detection zone and field of view (FOV). Orange triangle = Field of view and the white triangle = detection zone Amended from LtlAcorn official store (2023).

Yet, a major limitation among camera trap studies is a lack of consistency and detail in the reporting of camera trap methods (Burton et al. 2015). Studies often neglect to describe what make or model of camera trap was used, how many and where they were placed, what programmed settings were selected and, crucially, the decision‐making process behind those choices (Mann et al. 2015), leading to a direct loss of replicability. Published research on the differences between image capture rates between the same and different models is limited (but see Apps and McNutt 2018; Hughson et al. 2010; Palencia et al. 2022; Swann et al. 2004), making it difficult for researchers or conservation managers to make informed choices on equipment. This is disconcerting as research has shown that different or biased results can be obtained for species with different life history characteristics (i.e., body size, speed of movement) and as a result of camera trap specifications (i.e., detection zone, trigger speed, field of view, night vision range; Figure 1, Table 1) (Palencia et al. 2022).

**TABLE 1:** Camera trap specifications and how they affect efficacy.

Variable	Description	Effect
Detection zone	The zone covered by the camera's infrared sensor in which movement can be detected. (Figure 1). Composed of both detection angle and detection distance	A smaller detection zone angel can result in missed detections for faster moving animals but can result in the target animal being more centred and thus more likely that the whole body will be visible. A shorter detection distance will miss animals further away
Trigger speed	The delay between the detection of a target and the taking of the photo	A slower trigger speed can result in the image capture after the subject has passed (i.e., a false positive). However, a lower trigger speed (which can be adjusted on some models) can mean that the target has time to fully enter the frame before the photo is taken
Field of view (FOV)	The zone covered by the camera lens. (Figure 1)	A smaller FOV can result in a detected animal being outside or only partially in the shot. This increases the likelihood that the captured animal will be unidentified
Image quality/vision range	The resolution of the image and the distance at which the subject is discernible	Image quality is often adjustable (a higher quality requires more memory) but the higher the quality the more easily a distant subject can be identified
Low light image quality/ night vision range	The distance at which subjects can be discernible in low light and the degree of detail	A shorter night vision range can mean a subject is captured within the FOV but is obscured by darkness and is therefore unidentifiable. It is generally more variable than daylight range and can rarely be adjusted

Secondly, that camera detection zones (Figure 1 and Table 1), trigger speed (Table 1), field of view (Figure 1 and Table 1) and night vision range (Table 1) can affect the number and type of species captured (e.g., Palencia et al. 2022) (Table 1). Biases in the number or type of species captured, because of the settings used, could then have implications for the broader statistical analysis and approaches implemented as part of environmental impact assessments, conservation and wildlife management.

Species capture and identification can also vary among camera trap models as a result of external factors and study setup, even when cameras have the same programmed settings (e.g., Hughson et al. 2010; Moore et al. 2020; Palencia et al. 2022). Capture rates among different models can also be differentially affected by environmental factors such as humidity and extreme temperatures (Glover‐Kapfer et al. 2019; Swann et al. 2004). And thus, may differ in effectiveness depending on habitat/geographical location. Biases in capture rates increase when low population densities result in low detection probabilities. In this case small differences in capture rate can be greatly exaggerated (Hofmeester et al. 2021).

Given the rapid progress and improvement in camera trap design and function, a model that performs at what is considered a satisfactory standard at the time of purchase, may quickly perform at a standard below that of contemporary models. Furthermore, some older models of camera trap (> 10 years old) may incur bias in image captures when visible infrared flashes, or ‘glow’, disturb animals such as foxes (Meek et al. 2014), which may also bias image captures. Indeed, it is often neophobic, reclusive animals that are the target of camera trap surveys in research and management (e.g., Harley and Eyre 2024).

Beyond detection efficacy, there are practical factors that must be considered when selecting camera traps. Cost, for many researchers or conservation managers, is the primary decision factor when purchasing new or deciding to re‐use older camera trap models. This is particularly the case for increasingly popular applications of camera traps such as repeated annual wildlife surveys of nature reserves or in developing countries with limited funding, and research in this area is increasing (e.g., Agha et al. 2018; Akash et al. 2022; Cremonesi et al. 2021). Practitioners must beware that certain makes or models of camera traps may have high‐grade specifications in theory but perform worse in practice than those specifications would indicate (Palencia et al. 2022).

Some models may have the same detection rates, but are more susceptible to false positives, producing huge numbers of images and greatly increasing battery usage, cost and time taken for photo analysis (e.g., Meek et al. 2012). Other models may also be more durable or weather resistant or be compatible with weatherproof cases (Rovero et al. 2013). Durability is often a critical factor in model choice among research bodies or wildlife organisations that plan to deploy camera traps in potentially exposed areas or in tropical climates, over long durations of time or over multiple projects.

The wide range of factors that may affect camera trap performance, makes direct comparisons between studies unrealistic and disingenuous. Whilst, knowing differences in detection efficacy between camera trap models and settings could enable researchers to account for those differences, published comparison studies or efficacy tests of camera traps are scarce. Those studies that have been carried out are rarely performed in field conditions and tend to focus on active (baited/lured) as opposed to passive wildlife monitoring (e.g., Swan et al. 2014; Randler and Kalb 2018). Often these studies use artificial targets (people or domestic animals in a controlled setting), which do not reflect the situation in which researchers may wish to deploy camera traps although they may allow for more valid comparison (Apps and McNutt 2018; Randler and Kalb 2018; Swann et al. 2004; Yajima and Nakashima 2021).

The aim of this experiment was to compare the detection efficacy and false positive rates between different makes/models of camera traps to assess the influence of camera trap specifications and relatedly, price, on detection efficacy. Camera detection efficacy is a factor in overall detection probability of a target, which is generally defined as the combined probabilities of three events; (1) whether the target moves in front of the camera, (2) whether the animal then triggers the camera and (3) whether the target is present and identifiable in the image (Findlay et al. 2020; Hofmeester et al. 2019; Palencia et al. 2022).

For the in situ experiment, detection efficacy was compared across three camera trap models, at different price points and vintages, focusing on detection efficacy for medium‐large mammals across two feeding guilds, predators and herbivores. More specifically, the objectives were to (1) quantify how the total number of detections for predators and herbivores each differed between camera trap models; (2) determine at what time of day and under what conditions cameras were most effective; (3) contextualise the findings in terms of environmental impact assessment and emerging policies around net biodiversity gain. We expected disparity between models to be greatest for faster moving predators like pine marten than for slower moving large herbivores, in this case red deer, and for the number of true positives captured at night, as these scenarios require faster trigger speed and night vision range, factors that usually vary with camera trap quality.

## 2 Methodology

### 2.1 Site Description and Study Design

**FIGURE 2:**
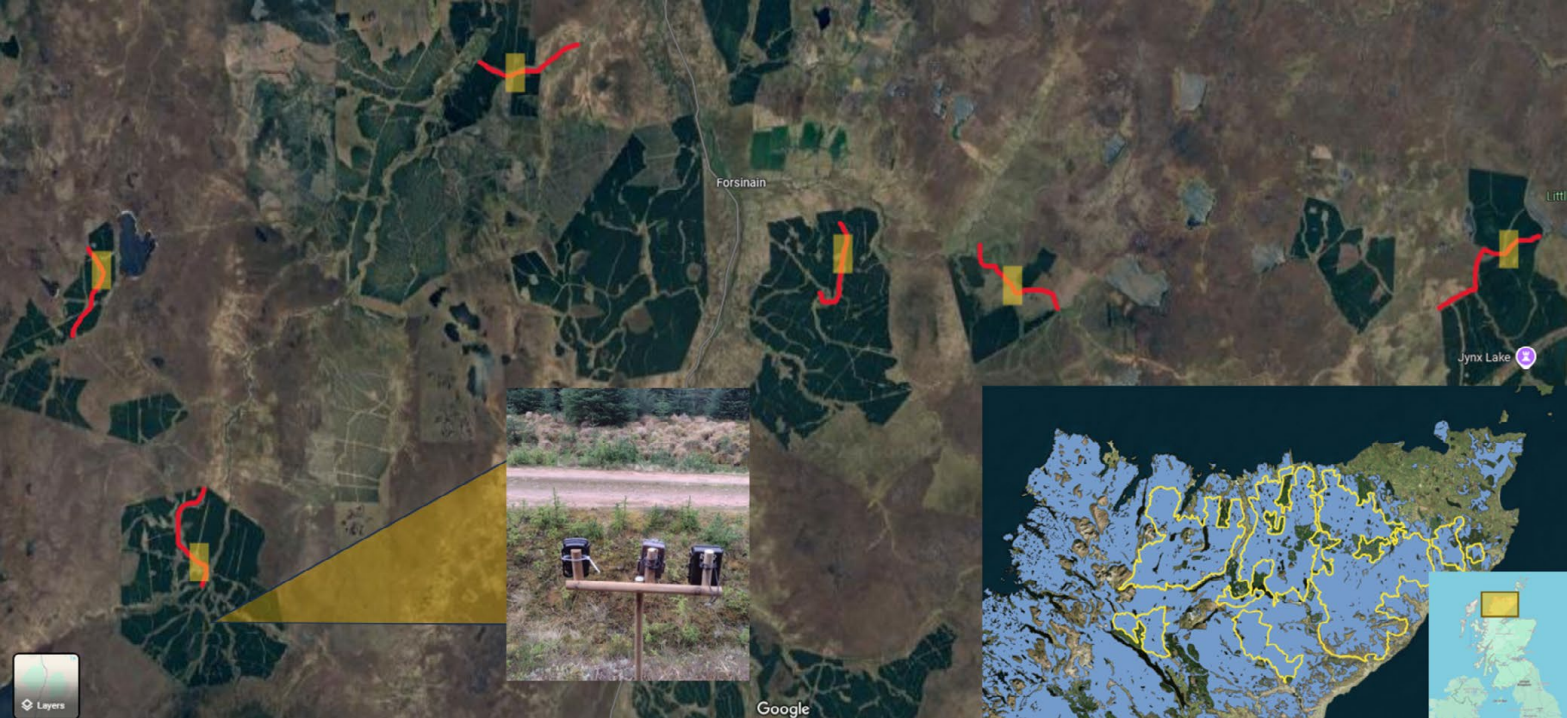
Arrangement of camera trap stations in Forsinard Flows nature reserve and the location and extent of the UNESCO Flow Country world heritage site in the United Kingdom (bottom left).

The study was carried out between February and April 2023 at Royal Society for the Protection of Birds (RSPB) Forsinard Flows National Nature Reserve, a protected area within the Flow Country peatlands of Caithness and Sutherland. The Flow Country is Europe's largest area of contiguous blanket bog and an UNESCO World Heritage Site covering an area of 4000 km^2^ (Lindsay et al. 1988) (Figure 2). However, it also includes large areas of non‐native conifer plantation established during the 1970s–80s. Mean daily maximum and minimum temperatures for March were 7.3°C and 4.2°C respectively, and average day length in March is 11 h 52 min (Atlas 2023; MetOffice 2023).

Our focal predator species were red fox and pine marten, which are typically more abundant in forestry plots compared to open peatland (unpublished data). We deployed six camera trap stations (one of each model) opportunistically along forestry roads. Previous work based on scat collection showed higher density of predators on roads than transects across the peatland, and predators have been shown to preferentially use roads where they are present in the landscape (Dickie et al. 2020). Therefore, we hoped this positioning would provide not only a higher chance of capturing predators but an improved view. We placed camera traps with a minimum distance to forest edge of between 152 m and 736 m (mean distance 134 mL). Forest plots varied in size from roughly 1.68 km^2^ to 4.4 km^2^.

Forest plots were chosen to be habitat replicates and to cover as wide an area within the available land as was possible, permissible by the landowner and practical to reach. Each forest plot consisted of mixed stands of *Picea sitchensis* and *Pinus contorta* separated by open peatland. There was a mean distance of 4.7 km (2.46–6.24 km) between camera trap stations (Figure 2).

To test the influence of camera trap model on detection efficacy we compared three camera trap models varying in price point and age.

The three camera traps models compared were:

1. the Apeman H45 (RRP £69.99/$85.51); the budget model, a low‐grade model used to monitor wildlife elsewhere in the landscape (https://uk.apemans.com/collections/trail‐camera/products/apeman‐trail‐camera‐h45#section_specification).
2. Bushnell HD (RRP £150/$183); the vintage model, an older camera model used for academic applications
  ~2015–2018 (https://service.bushnell.com/s/article/2013‐Trophy‐Camera‐Instruction‐Manual).
3. Browning Spec Ops HP5 (£199.99/$144.31); the high‐spec model, chosen specifically for the control as an example of a high‐grade, contemporary camera trap (https://browningtrailcameras.com/products/spec‐ops‐elite‐hp5).

**FIGURE 3:**
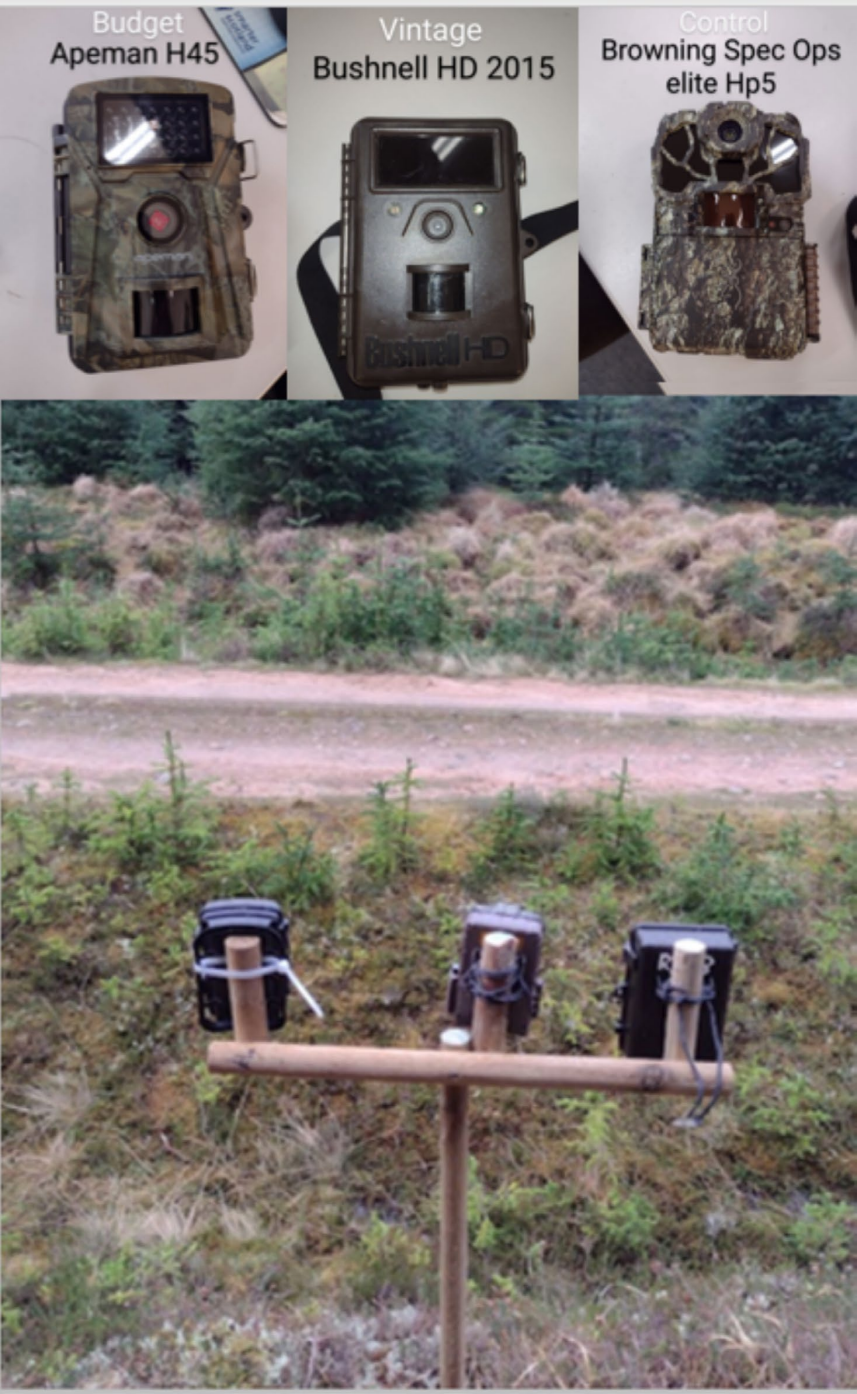
Camera trap setup in the field. From left; Apeman H45 (Budget), Browning Spec Ops HP5 (Control/High‐end), Bushnell HD (vintage).

One of each model was deployed continuously at the each of the six sites on the road edge and set to record for 50 days from February 17 to April 07, 2023. A duration of 50 days exceeds the minimum timeframe for accuracy recommended by Kays et al. 2020, however, our budget and the number of available forestry plots limited the number of cameras we could use so we increase the timeframe as a partial compensation. Camera traps were fixed along the top of a trident‐shaped wooden stake (Figure 3) designed for this study so that cameras could be attached to the stake as close to each other as possible. The design also allowed us to maximise overlap of FOV and detection zones and to ensure that all models were at the same height at each site. The stake was inserted in the bank 1–2 m from the road edge, approximately 1 m above ground level, which was a compromise between having a wide view of the road while being close enough to accurately identify small animals from the bank whilst being above vegetation Cameras were angled at 90° to the road, so that both the road and the bank on the opposite side were in view.

### 2.2 Camera Trap Settings

Each unit of all models was set to a two‐shot burst mode (i.e., two images taken in quick succession for each trigger), with a 1‐min minimum interval between triggers and to capture images at a quality of 8MP. Additional parameters were available in some models but were not used to enable a fair comparison (Table 2). For example, control cameras could be set to an image quality of 24 megapixels (MP), but this exceeded the maximum of 8 for vintage cameras, and while useful for other studies, it wasn't relevant to ours.

**TABLE 2:** Camera trap specifications by model. Refer to methods text for the actual set up used for consistency across all cameras.

Model	Apeman H45	Bushnell HD	Browning Spec Ops HP5
Year of purchase	2021	2012–13	2023
Price point (RRP)	£69.99/$85.51	£150/$183	£199.99/$248.82
Trigger speed (s)	0.5	0.6	0.1 (Programmable)
Recovery time (s)	NA	1	0.5
Field of view	73°	45°	53.7°
Detection range (m) Day	20 m	18 m	30 m
Detection range (m) Night	10 m	NA	27.5 m
Max resolution (Megapixels)	16 Mp (Programmable)	8 Mp	24 Mp (Programmable)
Infra‐Red Glow	No	Yes	No
Multi‐shot	< 3 (Programmable)	< 3 (Programmable)	< 8 (Programmable)
Memory capacity	32 GB	32 GB	64 GB
IP water/dust resistance rating	IP66* *IP rated as ‘dust tight’ and protected against heavy seas or powerful jets of water	NA	IP65 * *IP rated as ‘dust tight’ and protected against water projected from a nozzle

### 2.3 Image Analysis

Camera SD cards were retrieved at the end of the 50 days and the following data were collected for each camera: (1) total number of images; (2) total maintenance images (i.e., image taken as a result of setting up or maintaining cameras); (3) total false positives (i.e., images triggered but no visible subject, which include photo errors such as overexposed images or those that were all black or out of focus); (4) total positives (i.e., image triggered with subject present, maintenance images excluded); and (5) ‘recorded false negatives’. (occurrences when individuals entered the cameras' field of view but were not captured by any model). As it was not possible to calculate true false negatives, we subtracted total positives for a given camera from the total positives captured by the camera that recorded the highest value for that site. We recorded ‘0’ for the best camera and a net total false negatives for the other cameras at that site.

**FIGURE 4:**
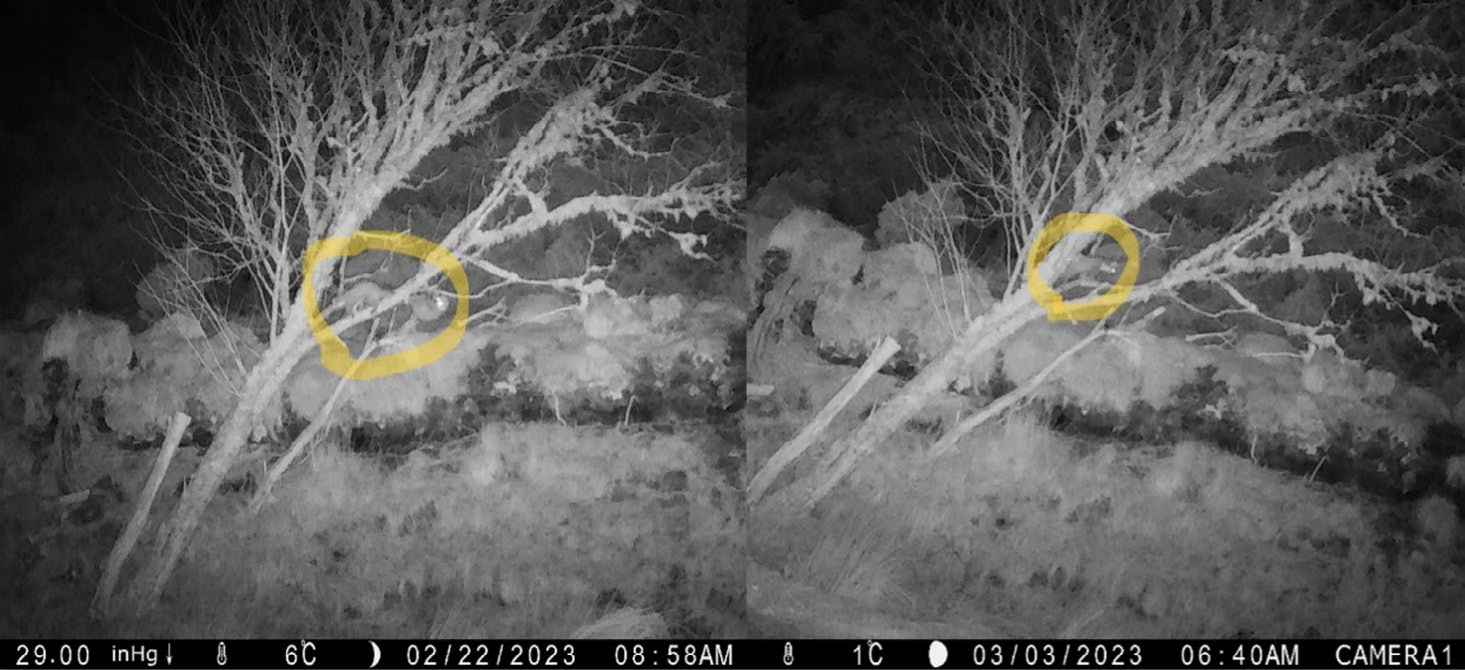
Two examples of a predator event, where two pine martens (left) and one pine marten (right) each count as a single predator event, provided they are separated in time by more than an hour.

Where an animal was not discernible to species level, it was not recorded. When species were recorded, they were aggregated to predator or herbivore grouping for the purpose of analysis. We devised the taxa specific term *predator event*, where a single predator event is used to describe any number of detections of at least one predator in an hour timeframe (Figure 4). If multiple predators are detected during this timeframe, they are counted as part of the same predator event, regardless of whether the individuals are the same or different. Events are only considered separate if there is a gap of more than an hour between detections. Relatedly, we chose not to attempt to identify species to the individual level in cases where they may be re‐entering the FOV in consecutive images as this was beyond the scope of this experiment, and because visual identification of individual unmarked animals is prone to inaccuracy (Sollmann et al. 2013).

Deer were not identified to species‐level as they are relatively similar in size and movement speed it was not relevant to our study to separate them. However, the study area contains mainly red deer ( *Cervus elaphus* ) and smaller numbers of roe deer ( *Capreolus capreolus* ). Other mammals were recorded to species. Birds were not identified to species‐level and were not included in the dataset analysed for the experiment.

To estimate the chance of detecting a species, or of it being present in a given study area at a given time, known as occupancy, we calculated a ‘Use of Area’ model, based on methods outlined by (Tobler et al. 2015). This is not a classic occupancy model, which would not have been compatible with our dataset due to the zero inflation and relatively few sites. Instead, a percentage value for predator use of area for each camera was calculated for each site and across all sites (*n* = 6) by taking the number of days a predator event was captured out of the total days (*n* = 50/site) that a model of camera was recording for each and all sites. We also calculated success rate for predators and deer that is, total predator or deer images as a percentage of the total images. And finally, naïve occupancy for all sites. Naïve occupancy is the proportion of sites where a species is detected during surveys but unlike typical occupancy modelling does not account for false absences. It is calculated by dividing the number of sites with at least one presence of a given species or taxa by the total number of sites surveyed.

## 3 Results

**FIGURE 5:**
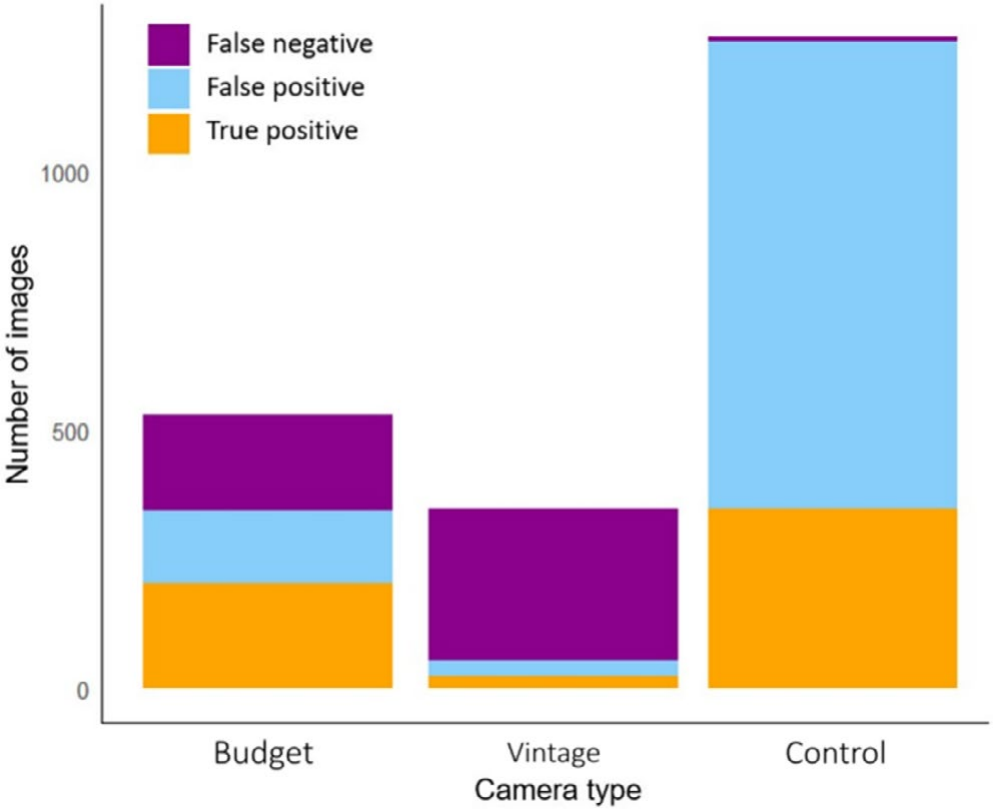
False positives, false negatives and true positives by camera trap type.

A total of 1768 images were captured (excluding maintenance and setup) over 300 (50 × 6) camera trap days including 579 (32.7%) true positives. The budget, vintage and control cameras respectively captured a total of 341 (60% of which positive), 55 (47%) and 1249 (29%) images (Figure 5). The control camera took three and 22 times the number of total images than the budget and vintage cameras, respectively. The total number of animals captured were 375 deer, 25 pine martens, 6 foxes, with the remaining images showing birds (*n* = 5) and otter *Lutra lutra* (*n* = 3), as well as 6 unidentifiable species (Figure 5). The budget and vintage cameras captured 27% and 4% of the total images captured by the control cameras. Net total recorded false negatives were 192, 274 and 8 respectively (Figure 5). Eight false negatives for the control cameras occurred where the target was out of the FOV of the control cameras as opposed to a direct missed detection. This false negative occurred at one site only (site 2) and was verified by the budget camera.

The percentage of positive images that contained predators for the budget, vintage and control cameras were 1.6%, 0% and 6.1% respectively. Mammalian predators triggered the control model in a total of 22 predator events. Predator capture success rate (total predator images as a percentage of the total images) was 1.8% (control) 1% (budget) and 0% (vintage). In four cases a predator was only visible in one of the two shots taken per trigger; in seven cases, which involved exclusively pine marten, more than one predator appeared in an image. Of the 22 predator events, 15 were pine martens, 6 foxes and 1 was an individual otter, which triggered both the control and budget cameras, appearing in both shots of the budget cameras burst mode. Only two predator events were captured in daylight, the otter and one pine marten.

**FIGURE 6:**
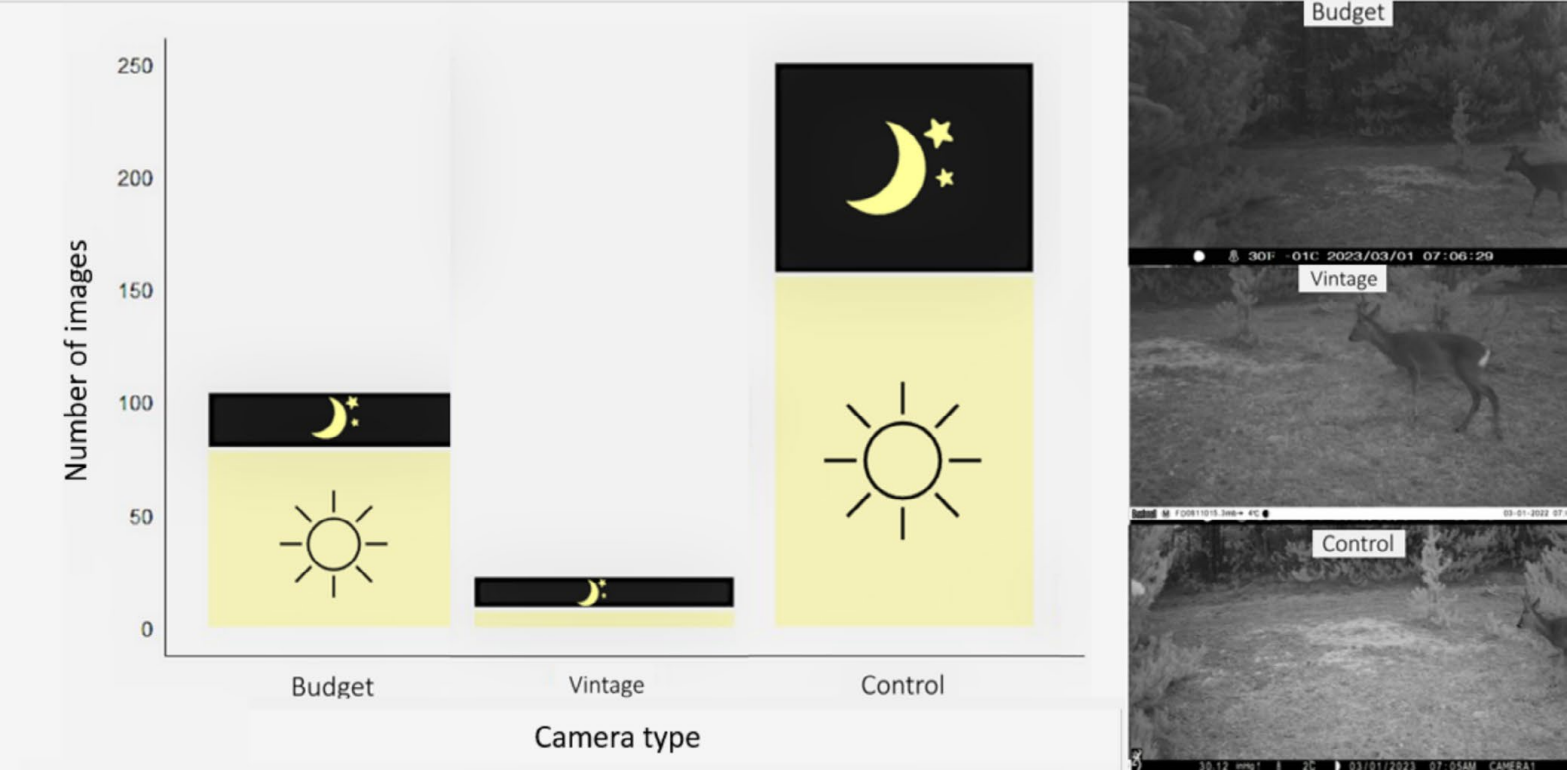
Left: Numbers of deer images (photographs) taken during day and night time for each camera trap type. Right: The same herbivore event as taken by each camera. Notice differences in FOV and image quality/range.

In total, 375 deer were recorded, 257 of which were captured by the control cameras. These contributed to a total of 364 herbivore events, of which the budget, vintage and control cameras captured 26%, 6% and 68%, respectively. Herbivore event success rate was 38% (budget) and 8% (vintage). Of individual deer 24%, 65% and 36% were captured during darkness by the budget, vintage and control camera, respectively (Figure 6). The control cameras captured all five birds.

For ‘use of area’ adapted from Tobler et al. (2015) we calculated that predators were recorded 26% of time across the study area by the control camera, 2% by the budget camera and 0% by the vintage camera. The greatest per site difference was at site five where a predator was recorded 16% of the time for the control camera and 0% for the budget and vintage cameras.

We calculated naïve occupancy for predators based on predator events, as no predators were recorded at the western sites (1–3), they had a naïve occupancy of zero. For the eastern sites (4–6) the control detected two fox events, two detected pine marten events, one otter event. That is, for site 4–6 a naïve occupancy of 66.7% for pine marten was recorded, 66.7% for fox also and 33.3% for otter. For the budget only site 6 detected a predator event so for sites 4–6 the budget camera produced a naïve occupancy of 33.3% for otter and predators overall and all other sites have a naïve predator occupancy of zero. For all sites combined the predator occupancy for the control camera was 50%, for pine marten otter and fox it was 33.3%, 33.3% and 16.7% respectively. The otter and the overall predator occupancy for the budget camera across all sites was 16.7%.

## 4 Discussion

### 4.1 Differences in Model Performances

We found that control cameras had notably higher detection rate than the other models for all species—not only for faster moving predators, where we expected the greatest disparity. We expected there to be no significant differences in the capture rate of large, relatively slow‐moving animals for example, deer, as in these cases a rapid trigger speed or wide detection zone would not be a key factor in the probability of detection (Hughson et al. 2010; Meek et al. 2014; Scheibe et al. 2008). However, control cameras captured 262% and 1223% more individual deer than did the budget and vintage models respectively, which is a greater difference demonstrated between models than that of any published camera trap comparison study.

In designing this study our initial hypothesis was that predator captures would be low for all cameras (< 1 capture/day) given the low densities of mammalian predators in the Flow Country, even within forestry plantations. We expected that false positives, and therefore likely total images, would be lower for control models on the assumption that higher quality detection systems might be better able to differentiate between live animals and moving vegetation or intense sunlight. In fact, the opposite occurred more frequently, in part due to higher in‐built sensitivity and also due to larger detection zones often causing a photo to be triggered before the animal entered the FOV (Hernandez et al. 1997; Swann et al. 2004; Wellington et al. 2014). However, unlike for the budget and vintage models in this experiment, sensitivity on the control camera can be adjusted manually. Trial and error are needed to determine the optimal sensitivity for maximising capture success while minimising false triggers, which can reduce battery life, consume memory card space, and increase the time and cost associated with sorting and analysing images (Meek and Pittet 2012).

The numbers of false negatives for deer and predators on both vintage and budget cameras was considerably greater than anticipated, based on specifications alone, for example, captures of deer in daylight that were not running was expected to be near equal given that they are large, generally relatively slow‐moving animals, that are most active during the day. Therefore, we did not expect the differences in detection parameters to be large enough to result in a pronounced difference in number of captures. While we expected there to be a difference in the number of predator captures, the specifications of the budget camera in particular did not appear to be too limiting or far from those of the control to produce such a large observed difference. It is possible that although differences in specifications may not be substantial, differences in overall build quality may be crucial to the efficacy of camera traps in this case.

At site five, the difference in the use of area score was substantial, a predator was recorded 16% of the time for the control camera and 0% for the budget and vintage cameras. Using this metric with data from the budget or vintage camera, the chance of detecting a predator in any given area would be extremely low. With a similar disparity for the naïve occupancy results. In application, this could mean numbers or activity of a seemingly often captured species such as an abundant herbivore may be greatly underestimated, potentially resulting in the implementation of ineffective conservation policies or conclusions. (Caravaggi et al. 2017; Nichols et al. 2011).

To a greater extent than predicted, our study confirmed that (a) there was a considerable disparity in predator captures between models and (b) that most captures occurred at night. We had anticipated that the greatest differences in capture rate would occur at night, as all predator captures by the budget camera during our wider study in these same peatland areas were taken at night. In several cases, the budget cameras captured a false positive image at night (no subject present) 1–3 min before or after when the control cameras captured a predator. This suggests that the budget cameras were sometimes triggered by a predator, but the image was taken before or after the animal had entered or left the frame or was out of night‐time range. This was potentially caused by an overly narrow detection zone, and as a result the animal moved through the frame before a photo is taken (Swann et al. 2004; Wellington et al. 2014). These events could also be an artefact of inadequate trigger speed or sensitivity (e.g., Findlay et al. 2020). As the control camera has a trigger speed five times faster than the budget camera (Table 2) fast‐moving animals at the edge of the frame are much more likely to be missed by the latter.

**FIGURE 7:**
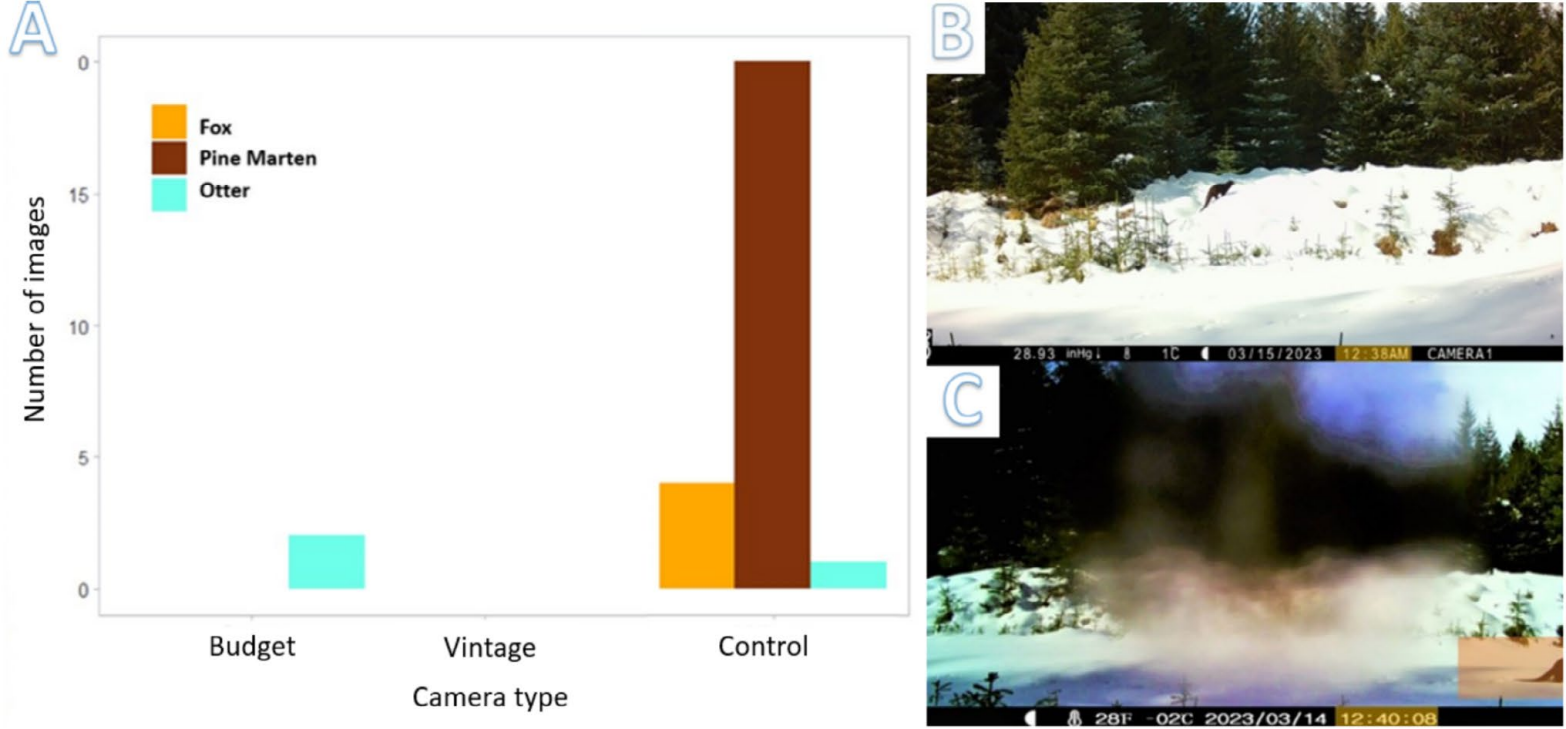
Comparison of one photo captured during the otter event by the control camera (A) and budget camera (C), the vintage camera failed to capture this event. B. Predator capture by individuals of each species for each camera type.

The results produced a substantially greater difference in positive images, particularly in the case of predators than predicted. That the only predator event (otter) captured by the budget camera was in daylight supports our assumption that limited night vision range is a factor in missed detections among the budget models. Although the otter was captured by the budget and control cameras over one event, the otter was in different locations within the FOV of the different models. According to the time stamp, the otter was captured 2 mins earlier by the control camera, on a snowbank that was just left of centre of the budget camera's FOV, suggesting the otter was moving within the detection zone for at least 2 minutes before a photo was taken (Figure 7B,C). However, the control camera failed to capture the otter in the second shot of the burst mode, instead producing a blank overexposed screen. The production of an overexposed image was not an isolated incident and occurred in 14 cases for the control camera during the experiment but was not produced by the budget nor the vintage camera. This phenomenon has been noted in other studies and is a result of the Infrared (IR) flash, flooding the image as a result of reflecting against an object, for example, the snowbank and is more common at higher flash intensities. (Rovero et al. 2013; Whytock et al. 2021). Otters have been demonstrated to be harder for camera traps to detect, as semi aquatic animals their fur is often wet, cooling their heat signature and making it more difficult for camera traps, which are triggered by heat differentials to capture them (Lerone et al. 2015). However, in this case the otter was at least 600 m from the nearest open water, giving it time to heat up.

The difference in the number of deer captured at night by each camera was notable. The budget cameras captured the lowest proportion of total deer at night as a total of deer images, often becoming blurred at distances greater than 3 m and indiscernible at distances greater than 10 m, depending on ambient light levels. The vintage cameras captured most deer at night 1–3 m from the camera lens. The control cameras captured deer in greater detail and at distances similar to, if not greater than in daytime, because of the infrared flash highlighting individuals at night. From our design, deer behind the forestry edge were never visible, so it is not known whether they succeeded in triggering the cameras. Taking this into consideration, both trigger speed and night vision range are key parameters for capturing animals, particularly nocturnal fast‐moving predators. Ascertaining the relative importance of trigger speed in detection probability is especially relevant to future camera trap studies, as previous work has shown that there is large variation in trigger speed among camera traps of the same make or even model (e.g., Driessen et al. 2017; Heiniger and Gillespie 2018; Hughson et al. 2010; Randler and Kalb 2018; Rovero et al. 2013). However, as all but one predator was captured at night, it is not possible to separately weigh the influence of these variables on detection probability of predators and, in general, positive captures were too low to enable quantifying the effect of night vision range alone on capture success.

Moreover, our data were not numerous enough to determine the influence of species size on detection probability, particularly as there were no large fast‐moving species or even deer captured while running instead of walking. The otter provided an example of a medium‐sized predator moving relatively slowly, apparently at less than running pace, but as only one otter was recorded it could not be used for any statistically significant comparison. Another factor to consider was that the roads in the study area are often cut into the peatland to reach the mineral layer below and as a consequence have deep sided banks running along their edges. It was necessary to place camera traps on these banks to get the largest field of view possible while being planted securely in a flat substrate. As a result, at certain sites, a portion of the road directly below the bank was out of each model's FOV, with the consequence that some smaller species or those that chose to run directly adjacent to the nearside road edge, may have been disproportionally missed by camera traps.

We did not experience any significant hardware issues over the course of the study, all models resisted waterlogging, animal attacks etc. However, the budget camera trap unlike the vintage and high‐end models does not have a hood over the camera lens to prevent drops on the lens (Figure 3) which obscured the image where they occurred (e.g., Figure 7C). Although beyond the scope of this study possible that some aspect of the study area contributed the size of disparity observed such as the high humidity, rainfall or wind in the Flow Country.

### 4.2 Implications for Camera Trap Studies Going Forward

Overall, our results demonstrated greater differences in total true positives and false negatives among camera trap models than any published study in both controlled and open environments (Apps and McNutt 2018; Driessen et al. 2017; Heiniger and Gillespie 2018; Palencia et al. 2022; Randler and Kalb 2018; Swann et al. 2004; Wellington et al. 2014). With significant data loss from the increased false negatives associated with using an older or lower quality camera trap model. Further, this data loss, or lack of detection, varied between target species, with lower grade models missing smaller, faster moving and nocturnal species in most cases, even where these species remained in or re‐entered the frame, for periods of up to 5 min. This is especially relevant as camera traps are often used as a low cost, non‐invasive way to monitor rare or elusive species, which are often carnivorous predators (e.g., Belbachir et al. 2015; Gerber et al. 2010; Rode et al. 2021). We suggest that researchers undertaking predator monitoring studies in future carefully consider the choice of using passive camera trap monitoring for their study.

Active monitoring with bait may be more likely to produce positive detections (Avrin et al. 2021; Buyaskas et al. 2020; Garrote et al. 2012; Glen et al. 2013; Meek et al. 2014; Randler et al. 2020; Rovero et al. 2013), provided it does not affect the experiment assumptions. However, baits and lures do increase the risk of attracting certain species, which may then artificially inflate their numbers/activity while repelling other species. Consequently, prey and more neophobic species that are often the target of passive monitoring studies may be less likely to be detected (e.g., Kruger et al. 2018; Kylmä 2018; Meek et al. 2014).

Importantly, our study demonstrates that researchers should avoid comparing numbers of images obtained from more than one camera trap model or from a mixture of newer and older versions of the same model within a study but also in analyses compiling data from multiple sites or studies. Where this is unavoidable, researchers should acknowledge the possible impact of camera trap model or specifications on the number of images captured and add these factors as a covariate in analyses. Here, we suggest that investment in high‐quality camera traps brings several benefits. We also recommend that if time, cost and labour constraints allow, then testing multiple models in field conditions prior to designing a wider study may be useful to determine which models are most effective at capturing the target animals for that study. As part of good practice, researchers should test at what range their camera trap model is most effective during both day and night and adjust camera trap orientation and subsequent statistical analysis accordingly. For example, by applying capture–recapture (CT SCR) models Distiller et al. (2020) or statistical procedures that deal with non‐detection biases (Royle et al. 2009; Twining et al. 2024). Additionally, studies should include a detailed description of the camera trap specifications and settings used when reporting results. We advise caution when reviewing studies or when researching experimental design, that authors avoid directly comparing studies that used different camera trap models or camera trap models older than 3 years and avoid making inferences about the abundance or activity of individuals relative to each other, other species, between sites or between geographical areas. For example, it would not be appropriate to assume that passive monitoring with a lower quality or older camera traps would yield predator activity or presence data that would be comparable with similar studies in similar environments but with higher quality camera traps.

Researchers, developers, conservationists and wildlife managers should be aware of making assumptions about the abundance or activity of certain species in an area based on passive camera trap monitoring alone, especially when camera trap specifications are not provided. And should work toward integrating camera traps alongside other survey types for example, scat counts or thermal imaging. Which can be applied alongside camera trap data can in analyses for example, using integrated community occupancy models (ICOMs) (Doser et al. 2022). This is particularly relevant in the context of developments (e.g., wind farms) where Environmental Impact Assessment is needed and where suitable habitat management plans should be implemented—which require establishing baseline biodiversity data. In the most serious case, developers may unknowingly impact protected species due to assumptions based on flawed camera trap data risking threatening protected species, litigation and regulatory intervention. For example, if the activity of known nest predators is concluded to be low based on camera trap data, then conservationists may erroneously reintroduce a threatened bird species compromising the reintroduction effort. Conversely in a predator control scenario, camera trap data might suggest predator activity is too low to merit the continuation of control measures, which may be crucial to ecosystem functioning.

To avoid this, we would advise that the absence or activity of a species cannot be assumed based on the lack of images derived from camera trap surveys and encourage the application of statistical analyses developed to account for non‐detection biases for example, Twining et al. (2024). It would be useful for policy makers and developers to develop guidance on the minimum standard information required for meaningful interpretation of data collected from camera trap survey, possibly using mitigation strategies where high‐end equipment is unavailable (Table 3). When modelling the results of a passive camera trap study, researchers should account at the minimum for the effect of time of day, the model used, the size, behaviour etc. of the species targeted.

**TABLE 3:** Camera trap type chosen and mitigations in order of efficacy.

Camera type	Primary mitigation	Secondary mitigation	Tertiary mitigation	Other comments
Budget camera (< $100)	Avoid using if possible	Use to target slower moving and/or diurnal animals	Use more than one camera per site	Compare with studies using same model or for the exact specifications and known faults in statistical analysis
Older model	Avoid using if possible. Target slower moving and diurnal animals	Increase camera trap number, potentially using more than one camera trap per site	Use baits or lures	

A principal aim of environmental impact assessment (EIA) is to gain a precise understanding of the ecological context which a proposed development may affect. Relatedly the objective of biodiversity net gain is to ensure wildlife habitats are left in a measurably better state than they were before the development (Department for Environment, Food, and Rural Affairs 2024). If tools for measuring biodiversity such as camera traps provide inconsistent results, then developers may assume for example, that a habitat is of lesser biodiversity value than it is or that a specific action to increase biodiversity net gain value has been more or less effective than it was in reality.

## 5 Conclusion

This experiment has demonstrated a greater difference in detection efficacy among camera trap models than any published comparison so far. The extent of the disparity in positive images between an industry standard, high‐end camera trap and cheaper or older models should help researchers and conservationists make informed decisions about design and camera choice. Where accuracy is required, then budget and even high‐quality camera traps that are dated more than 2–3 years may not be suitable, even when the specifications appear to closely match current high‐end models. This is especially the case where the subjects are fast‐moving or nocturnal animals. If unable to use high‐end models due to cost restrictions, practitioners should explore the use of baits or lures, increase the number of lower cost camera traps at each site and add camera model or age as a covariate in statistical analyses. Popularising the use of contemporary high‐quality cameras with similar specifications should help to standardise camera trap‐based research and conservation and contribute toward the progress of conservation science by enhancing reproducibility and rigour.

## Author Contributions

**R. McHenry:** conceptualization (lead), formal analysis (lead), investigation (lead), methodology (lead), software (equal), writing – original draft (lead), writing – review and editing (equal). **L. J. Mitchell:** conceptualization (equal), formal analysis (equal), investigation (equal), methodology (equal), supervision (equal), writing – review and editing (equal). **C. Marshall:** supervision (equal), writing – review and editing (equal). **J. Smart:** funding acquisition (equal), supervision (equal), writing – review and editing (equal). **A. L. de Raad:** supervision (equal), writing – review and editing (equal). **R. Andersen:** formal analysis (lead), funding acquisition (lead), software (equal), supervision (lead), writing – review and editing (equal).

## Conflicts of Interest

The authors declare no conflicts of interest.

## Supporting information


**Appendix S1.** The ‘use of area’ model was calculated by counting the total number of dates on which a predator was captured and dividing by the total 50 days to get a percentage use of area for each and all sites.

## Data Availability

Data supporting this study are included within the article and/or supporting material. For further information contact Robert McHenry 21020976@uhi.ac.uk.
